# MicroRNA-376a Regulates 78-Kilodalton Glucose-Regulated Protein Expression in Rat Granulosa Cells

**DOI:** 10.1371/journal.pone.0108997

**Published:** 2014-10-03

**Authors:** Masayuki Iwamune, Kazuto Nakamura, Yoshikazu Kitahara, Takashi Minegishi

**Affiliations:** 1 Department of Obstetrics and Gynecology, Gunma University Graduate School of Medicine, Maebashi, Gunma, Japan; 2 Department of Gynecology, Gunma Prefectural Cancer Center, Oota, Gunma, Japan; China Agricultural University, China

## Abstract

The 78-kilodalton glucose-regulated protein (GRP78) is a molecular chaperone that assists in protein assembly, folding, and translocation. Recently, our laboratory reported that GRP78 regulates the expression of luteinizing hormone-human chorionic gonadotropin receptor (LHR) in the early stage of corpus luteum formation. In this study, we investigated whether microRNAs (miRNAs), which post-transcriptionally regulate mRNA, are involved in the regulation mechanism of GRP78 in the ovary. A miRNA microarray was performed to analyze the overall miRNA expression profile, and the results indicated that 44 miRNAs were expressed highly after ovulation was induced. The results from a bio-informative database analysis and *in vitro* granulosa cell culture studies led us to focus on rno-miR-376a for further analysis. In both *in vivo* and *in vitro* studies, rno-miR-376a levels increased 12 h after human chorionic gonadotropin (hCG) administration. To elucidate whether rno-miR-376a induced mRNA destabilization or translational repression of GRP78, rno-miR-376a was transfected into cultured granulosa cells, resulting in decreased GPR78 protein levels without an alteration in GRP78 mRNA levels. To confirm that rno-miR-376a binds to GRP78 mRNA, we cloned the 3′-end of GRP78 mRNA (nucleotides 2439–2459) into a reporter vector that contained a Renilla luciferase coding region upstream of the cloning site. The luciferase assays revealed that rno-miR-376a bound to the 3′-end of GRP78 mRNA. From these data, we conclude that rno-miR-376a potentially negatively regulates GRP78 protein expression through translational repression at an early stage transition from the follicular phase to luteinization.

## Introduction

In females, ovulation—the release of a mature and fertilizable oocyte—is an essential process in the establishment of pregnancy. Follicle stimulating hormone (FSH) is a key factor that induces luteinizing hormone-human chorionic gonadotropin receptor (LHR) expression in the granulosa cells of pre-ovulatory follicles, whereby an LH surge triggers ovulation followed by corpus luteum formation [1], [2], [3]. Ovulation provokes a dramatic transformation of ovulated follicle to form the corpus luteum, which in turn induces synthesis of progesterone in order to sustain pregnancy. This indicates that granulosa cells are under stress, which has led to ovulation being considered an inflammation-like phenomenon [4], [5].

When a cell is exposed to a variety of environmental and physiological stresses, the function of the endoplasmic reticulum (ER) is disturbed, and unfolded proteins accumulated in the ER [6]. In the ER, molecular chaperones are responsible for proper protein folding and for preventing aggregation of unfolded-intermediates, which could lead to cell death [7]. Recently, our laboratory determined that the 78-kilodalton glucose-regulated protein (GRP78), an ER-associated molecular chaperone that assists in proper protein folding to execute primary protein maturation in the ER [8], [9], is involved in the recovery of LHR after its down-regulation [10]. Although GRP78 is known be an important molecule for the upregulation of LHR, the precise mechanism underlying the regulation of GRP78 expression in the ovary has not been fully elucidated.

MicroRNAs (miRNAs) are non-coding RNAs (approximately 22 nucleotides) that regulate gene expression by binding to the 3′-untranslated regions of target mRNAs to induce the degradation of target mRNAs or to induce translational repression [11]. Within the past decade, miRNAs have been recognized as important regulators in many biological and cellular processes, such as cell proliferation, differentiation, apoptosis, and tumorigenesis [12], [13], [14]. Although the functions of miRNAs have not been elucidated fully, recent emerging evidence demonstrates that miRNAs are involved in ovarian follicular and luteal functions [15], [16], [17], [18]. We have also reported that miR-136-3p targets LHR mRNA to induce the transient downregulation of LHR mRNA in granulosa cells after ovulation [19]. This result prompted us to search for miRNAs involved in the regulation of GRP78 expression.

In the following experiments, we profiled the miRNAs that were expressed in PMSG-primed rat ovaries in which an injection of hCG induced ovulation. From that data and data obtained from a bio-informatics database, we focused on rno-miR-376a, which potentially binds to GRP78 mRNA, and characterized the function of rno-miR-376a in cultured granulosa cells.

## Materials and Methods

### Ethic Statement

All procedures involving animals followed ethical principles according the NIH Guide for Care and Use of the Laboratory Animals and were approved by the Gunma University Animal Care and Use Committee (Permit Number: 12–011).

### Hormones and reagents

Recombinant FSH and purified hCG were supplied by Dr. A. Parlow and the National Hormone and Peptide Program (National Institute of Diabetes and Digestive and Kidney Disease, National Institutes of Health, Torrance, CA, USA). Dulbecco modified Eagle medium (DMEM), DMEM/Ham's Nutrient mixture F-12, diethylstilbestrol (DES) and β-Estradiol-Water Soluble were purchased from Sigma Chemical Co. (St. Louis, MO, USA). Gentamicin sulfate and Fungizone were purchased from Invitrogen Corp. (Carlsbad, CA, USA).

hCG for the *in vivo* study was provided by Schering-Plough Corp. (Osaka, Japan), and PMSG was provided by Asuka Pharmaceutical Co., Ltd. (Tokyo, Japan).

### Animals

Female 21-day-old Wistar rats (Japan SLC, Inc., Hamamatsu, Japan) were housed in a temperature- and light-controlled room (12 h light, 12 h dark cycle; lights on at 0600 h) with food and water provided *ad libitum*.

For the *in vivo* study, 21-day-old female rats received an injection of PMSG (30 IU, subcutaneous [sc]), followed by an injection of hCG (20 IU, sc) 48 h later. We then sacrificed the rats by decapitation to remove the ovaries at each time point after the injection of hCG. The ovaries were stored immediately in RNAlater Tissue Collection solution (Applied Biosystems, Foster City, CA, USA).

For the *in vitro* study, 21-day-old female rats received DES injections (2 mg, sc) for 4 d, and the ovaries were removed for use in primary granulosa cell culture, as described below.

### Primary rat granulosa cell culture

Granulosa cells were obtained from DES-primed rats. Both ovaries were removed from the rats, and the granulosa cells were released by puncturing the follicles with a 26-gauge needle. The granulosa cells were washed and collected by brief centrifugation, and cell viability was determined by trypan blue exclusion. The granulosa cells were then cultured in DMEM/Ham's nutrient mixture F-12 supplemented with 20 mg/L gentamicin sulfate, 500 µg/L Fungizone, and 1 g/L bovine serum albumin (BSA) on collagen-coated plates or dishes in a humidified atmosphere containing 5% CO2 and 95% air at 37°C.

### miRNA microarray

Rat ovaries were removed from PMSG/hCG-treated rats to analyze miRNA expression on a microarray. The miRNA microarray expression profiling was performed by TaKaRa Bio Inc. using the Agilent Technologies Rat miRNA Microarray 8×15K (Santa Clara, CA, USA), which contains 350 rat miRNAs listed in the Sanger miRBase v.10.1. Total RNA, including miRNAs, was isolated from the PMSG/hCG-treated rat ovaries using the FastPure RNA Kit (TaKaRa Bio Inc., Otsu, Japan) according to the manufacturer's protocol. A quality check of the total RNA was performed using an Agilent 2100 Bioanalyzer (Agilent Technologies). The labeling and hybridization of the total RNA samples were performed using an Agilent miRNA Microarray System (Agilent Technologies). The microarray results were extracted using Agilent Feature Extraction software, and the data were analyzed by TaKaRa Bio Inc.

### miRNA target prediction

Target miRNAs that bound to rat GRP78 mRNA were predicted using MicroCosm Targets (http://www.ebi.ac.uk/enright-srv/microcosm/htdocs/targets/v5/; hosted by the European Bioinformatics Institute) based on the miRNA microarray results.

### miRNA expression profiling of rat ovaries and rat granulosa cells using TaqMan miRNA assays

Total RNA, including miRNAs, was isolated from rat ovaries or rat granulosa cells using the mirVana miRNA Isolation Kit (Ambion Inc., Austin, TX, USA) according to the manufacturer's protocol. The concentration of the total RNA was measured using a Nanodrop-1000 spectrophotometer (Thermo scientific, Wilmington, DE, USA). The total RNA was then reverse transcribed using specific RT primers from a TaqMan MicroRNA Assay Kit (rno-miR-144: 197375, rno-miR-376a: 001069, rno-miR-451: 001141, and 4.5S RNA(H): 001716 as an internal control; Ambion Inc.). Single-stranded cDNA was synthesized from 10 ng of total RNA in a 15-µL reaction volume using the MicroRNA Reverse Transcription Kit (Applied Biosystems), according to the manufacturer's protocol. The reactions were incubated for 30 min at 16°C, 30 min at 30°C, and 5 min at 85°C in a thermal cycler. Real-time RT-PCR was performed using sequence-specific primers from the TaqMan MicroRNA Assay Kit according to the manufacturer's instructions. The reactions were performed using an ABI PRISM 7000 sequence detection system (Applied Biosystems) in a 20-µL reaction volume at 95°C for 10 min, followed by 40 cycles of 95°C for 15 s and 60°C for 60 s. All of the reactions were tested in triplicate. We determined the threshold cycle (Ct) values for each reaction, and their means were used to determine the relative miRNA expression levels using the ΔΔCt method. Additionally, we calculated the percentage of rat GRP78 mRNA or miRNA expression using the ΔΔCt values for each reaction as follows:



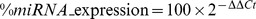



These values were used to calculate, and the relative expression of GRP78 or each miRNA was normalized to the expression of hCG 0 h in the control, which was set at 1.

### Expression of GRP78 mRNA expression in rat ovaries or rat granulosa cells using TaqMan Gene Expression Assays

Total RNA was isolated from rat ovaries or rat granulosa cells, and the concentrations were measured as described above. The isolated RNAs (2 µg of each sample) were treated with DNaseI (Invitrogen) to eliminate residual genomic DNA. These RNAs were reverse transcribed using random primers, 10 mM deoxynucleoside triphosphate mix, and SuperScript III reverse transcriptase (Invitrogen) according to the manufacturer's protocol. The reactions were incubated for 5 min at 25°C, 60 min at 50°C, and 15 min at 70°C in a thermal cycler. To remove complementary RNA, RNase H was added to the cDNAs, and the reactions were incubated for 20 min at 37°C. Real-time RT-PCR was performed using TaqMan Gene Expression Assays (GRP78-Hspa5: Rn00565250_m1, Eukaryotic 18S rRNA: Hs99999901_s1 as an internal control; Applied Biosystems) according to the manufacturer's instructions. Rat GRP78 mRNA expression levels were measured in a similar manner as in the miRNA assays.

### Transfection with Pre-miR-376a miRNA (precursor) and Anti-miR-376a miRNA (inhibitor)

Primary rat granulosa cells (8.0–10×10^4^ cells obtained from DES-primed rats) were cultured in 24-well tissue culture plates with serum-free medium. Twenty-four hours after seeding, the cells were incubated with FSH (30 ng/mL) and estradiol (10 nM) for 48 h. The cells were then transfected with Pre-miR-376a (precursor) or Anti-miR-376a (inhibitor) (50 nM each) purchased from Ambion (product IDs: PM10504 and AM10504, respectively) using siPORT NeoFX Transfection Agent according to the manufacturer's protocol. Twelve hours after transfection, hCG (30 ng/mL) was added to the culture medium to induce the downregulation or upregulation of GRP78 mRNA.

To evaluate the effects of precursor and inhibitor on GRP78 mRNA expression, we performed real-time RT-PCR using TaqMan gene expression assays 12 h after hCG treatment and compared the results with the mock-transfected cells. All of the reactions were tested in triplicate. Rat GRP78 mRNA expression was measured in a similar manner as in the miRNA assays.

### Western blot analysis

Primary rat granulosa cells (150×10^4^ cells obtained from DES-primed rats) were cultured in 3.5-cm tissue culture dishes with serum-free medium. Twenty-four hours after seeding, the cells were incubated with FSH (30 ng/mL) and estradiol (10 nM) for 48 h. The cells were then transfected with Pre-miR Negative control #1 (product IDs: AM17110), precursor or inhibitor (50 nM each) purchased from Ambion, using Lipofectamine 2000 Transfection Reagent according to the manufacturer's protocol. Twelve hours after transfection, hCG (30 ng/mL) was added to the culture medium.

The cells were lysed in radio-immunoprecipitation assay buffer (150 nM NaCl, 50 mM Tris, 1 mM EDTA, 1% NP-40, 0.5% sodium deoxycholate, 0.1% SDS, pH 7.4) containing proteinase inhibitors (PMSF, pepstatin A, and leupeptin). The protein lysates were resolved on sodium dodecyl sulfate gels and electrophoretically transferred to a polyvinylidene difluoride (PVDF) membrane. After blocking, the expression of GRP78 was determined using a rabbit anti-GRP78 antibody (1∶1000; AnaSpec, Inc.) and a horseradish peroxidase-conjugated goat anti-rabbit immunoglobulin G (IgG) antibody (1∶40000; Bio-Rad Laboratories, Inc.). The proteins bands were visualized using enhanced chemiluminescence (Immobilon Western; Millipore). Luminescence detection was quantified by scanning the films with a CCD camera and analyzing the digitized data using the NIH ImageJ software.

### Reporter vectors and DNA constructs

To identify the rno-miR-376a-binding site at the 3′-end of GRP78 mRNA, we generated a direct-match miRNA target site and cloned the insert into the multiple cloning site in the luciferase reporter vector from the pMIR-REPORT miRNA Expression Reporter Vector System (Applied Biosystems). The sense and antisense strands of the oligonucleotides were annealed by adding 2 µg of each oligonucleotide to 46 µL of annealing solution (100 mM potassium acetate, 30 mM HEPES-KOH, pH 7.4 and 2 mM magnesium acetate) and incubating at 90°C for 5 min and then at 37°C for 1 h. The annealed oligonucleotides were digested with *Hind*III and *Spe*I and ligated into the *Hind*III and *Spe*I restriction sites of pMIR-REPORT vector. The sequences of the inserts were confirmed by sequence analysis using a PRISM 3100 genetic analyzer (Applied Biosystems).

The following oligonucleotides were used in this studies: (1) *miR-376a*,

5′-aatgcactagtACGAGGATTTTCCTCTACGATaagcttaatgc-3′ and

5′-gcattaagcttATCGTAGAGGAAAATCCTCGTactagtgcatt-3′, and

(2) rno-miR-376a-binding site sequence at the 3′-end of GRP78 mRNA,

5′-aatgcactagtATGGTAGAAAAAAGTTCCTACaagcttaatgc-3′ and

5′-gcattaagcttGTAGGAACTTTTTTCTACCATactagtgcatt-3′

### Luciferase assay

We transfected HEK293 cells with 200 ng of each vector (pMIR-REPORT luciferase vectors, as described above, and the pMIR-REPORT βgal vector as a control for transfection normalization) and 50 nM precursor or inhibitor using Lipofectamine 2000 Transfection Reagent according to the manufacturer's protocol. To measure the luciferase activity, we harvested the cells 24 h after transfection and conducted the assay using the pMIR-REPORT miRNA Expression Reporter Vector System (Applied Biosystems).

### Data analysis

The microarray data were first filtered by subtracting the control probe data. To identify miRNAs, a one-way ANOVA was performed for differentially expressed miRNAs between four groups (hCG 0 h, hCG 6 h, hCG 12 h, hCG 24 h). False discovery rates (FDR) were assessed using the Benjamini and Hochberg method. Hierarchical clustering was then performed using the complete linkage method.

Comparisons between groups were performed using one-way ANOVA followed by Dunnett's multiple comparison test. The data represent the mean ±SE from at least three independent experiments. A value of *P*<0.05 was considered significant.

## Results

### miRNA microarray and target prediction

We utilized a miRNA microarray analysis to identify miRNAs that were expressed in rat ovaries. Based on the differentially expressed miRNAs, a cluster analysis was executed to generate subgroups characterized by the expression patterns in four groups (hCG 0 h, hCG 6 h, hCG 12 h, and hCG 24 h) (Fig. S1). A total of 44 miRNAs were found to differ significantly in response to hCG treatment, as measured using the Benjamini and Hochberg method, suggesting that these miRNAs potentially affected the expression of GRP78. From these 44 miRNAs, we focused on 23 miRNAs that increased 6 h after hCG treatment and then decreased by 24 h (Table 1), and 19 miRNAs that decreased 6 h after hCG treatment and then increased by 24 h (Table 2). The results of the bioinformatics database inquiry (MicroCosm, http://ebi.ac.uk/) revealed that rno-miR-144, rno-miR-376a, and rno-miR-451 can bind to the 3′-UTR of GRP78 mRNA (from bp 2439–2459) and negatively regulate GRP78 expression.

**Table 1:** List of differentially expressed (upregulated) miRNAs in rat ovaries following treatment with hCG for 6 h.

miRNA	Fold change	*q*-value	miRNA	Fold change	*q*-value
*rno-miR-222*	1.28	0.001	*rno-miR-410*	1.60	0.039
*rno-miR-379*	1.45	0.014	*rno-miR-541*	1.46	0.031
*rno-miR-376b-3p*	1.46	0.014	*rno-miR-132*	1.52	0.005
*rno-miR-411*	1.68	0.030	*rno-miR-146b*	1.39	0.020
*rno-miR-136-3p*	1.58	0.010	*rno-miR-125b-3p*	1.50	0.043
*rno-miR-434*	1.56	0.042	*rno-miR-99a-3p*	1.35	0.042
*rno-miR-136-5p*	1.43	0.020	*rno-miR-341*	1.39	0.039
*rno-miR-431*	1.37	0.029	*rno-miR-652*	1.25	0.081
*rno-miR-376a*	1.50	0.014	*rno-miR-181d*	1.09	0.358
*rno-miR-154*	1.30	0.038	*rno-miR-181c*	1.11	0.231
*rno-miR-127*	1.31	0.019	*rno-miR-503*	1.05	0.648
*rno-miR-21-3p*	2.30	0.019			

**Table 2:** List of differentially expressed (upregulated) miRNAs in rat ovaries following treatment with hCG for 12 h.

miRNA	Fold change	*q*-value	miRNA	Fold change	*q*-value
*rno-miR-185*	0.97	0.002	*rno-miR-33*	1.11	0.010
*rno-miR-34a*	1.09	0.010	*rno-miR-22*	1.05	0.010
*rno-miR-144*	1.22	0.030	*rno-miR-140*	1.04	0.002
*rno-miR-451*	1.21	0.030	*rno-miR-140-3p*	1.00	0.001
*rno-miR-7a*	1.32	0.030	*rno-miR-193*	1.13	0.010
*rno-miR-425*	1.10	0.010	*rno-miR-877*	1.07	0.040
*rno-miR-28*	1.00	0.010	*rno-miR-29b*	1.10	0.008
*rno-miR-29a*	1.06	0.030	*rno-miR-24-2-3p*	1.23	0.010
*rno-miR-18a*	1.20	0.010	*rno-miR-21*	1.18	0.010
*rno-miR-27a*	1.04	0.030			

### miRNA expression in rat ovaries

**Figure 1:**
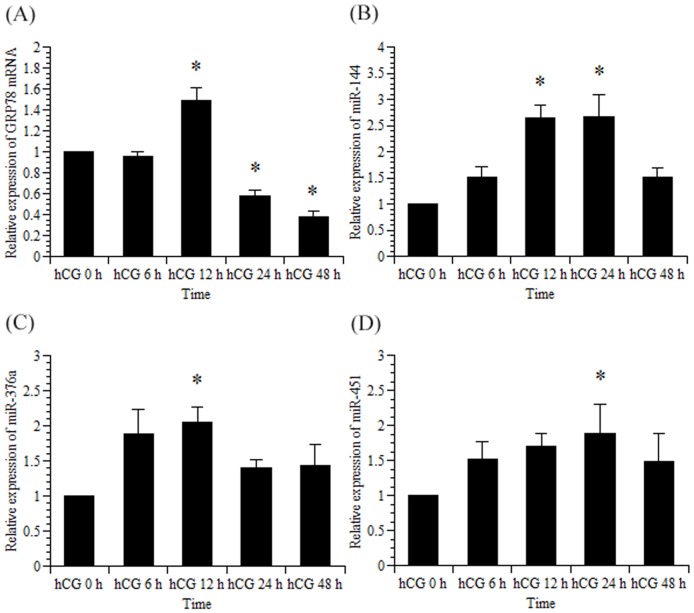
Time course of rat GRP78 mRNA, rno-miR-144, rno-miR-376a, and rno-miR-451 expression in rat ovaries induced by PMSG and hCG. Female 21-day-old rats injected subcutaneously with PMSG (30 IU/rat), followed by hCG (20 IU/rat) 48 h later, were sacrificed at the indicated times. The ovaries were removed, and total RNA was isolated. Rat GRP78 mRNA (A), rno-miR-144 (B), rno-miR-376a (C), and rno-miR-451 (D) expression levels were measured using real-time RT-PCR as described in the Materials and Methods. The amount of rat GRP78 mRNA, rno-miR-144, rno-miR-376a, and rno-miR-451 in the hCG 0 h group was set at 1. Data were normalized to 18S rRNA (for GRP78 mRNA) and 4.5S RNA(H) (for rno-miR-144, rno-miR-376a, and rno-miR451) levels in each sample and represent the mean ±SE of three independent experiments. *, significantly different from the control value at hCG 0 h, *P*<0.05.

In both *in vivo* and *in vitro* experiments, we previously demonstrated that GRP78 mRNA levels peaked while LHR mRNA was downregulated by hCG and that the increase of LHR protein levels was dependent on the increment of GRP78 protein [10]. Therefore, we measured the expression of GRP78 mRNA, rno-miR-144, rno-miR-376a, and rno-miR-451 using real-time RT-PCR to examine their expression patterns in the context of LHR downregulation (Fig. 1). Consistent with previous studies, GRP78 mRNA upregulation peaked 12 h after the administration of an ovulatory dose of hCG and subsequently decreased. In a similar manner, rno-miR-144 and rno-miR-376a expression peaked 12 h after the hCG treatment, and rno-miR-451 expression peaked 24 h after the hCG treatment.

### miRNA expression in rat granulosa cells

**Figure 2:**
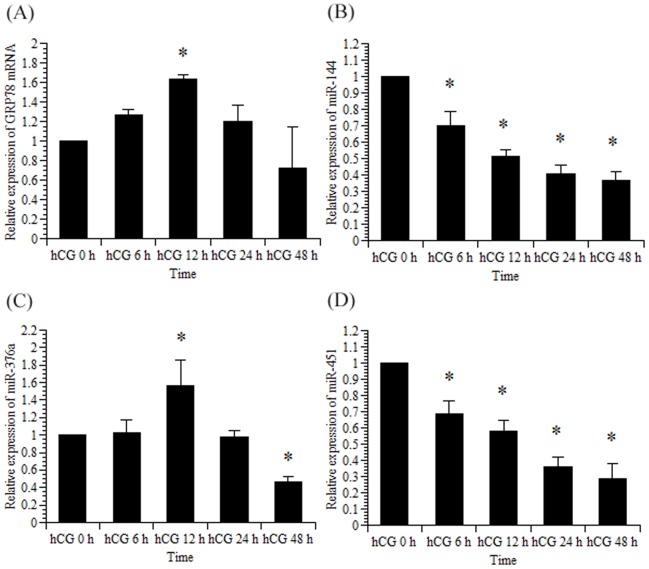
Rat GRP78 mRNA, rno-miR-144, rno-miR-376a, and rno-miR-451 expression in primary rat granulosa cells induced by FSH and hCG. Primary rat granulosa cells were prepared, and the indicated reagents were added to the medium after 24 h of culture. Cells were then incubated with FSH (30 ng/mL) and estradiol (10 nM) for 48 h. Subsequently, hCG (30 ng/mL) was added to the culture medium, as described in the Materials and Methods. Total RNA was isolated, and GRP78 mRNA (A), rno-miR-144 (B), rno-miR-376a (C), and rno-miR-451 (D) expression levels were measured using real-time RT-PCR as described in the Materials and Methods. The amounts of GRP78 mRNA, rno-miR-144, rno-miR-376a, and rno-miR-451 in the hCG 0 h group were set at 1. Data were normalized for 18S rRNA (for GRP78 mRNA) and 4.5S RNA(H) (for rno-miR-144, rno-miR-376a, and rno-miR-451) levels in each sample and represent the mean ±SE of 3 independent experiments. *, significantly different from the control value at hCG 0 h, *P*<0.05.

**Figure 3:**
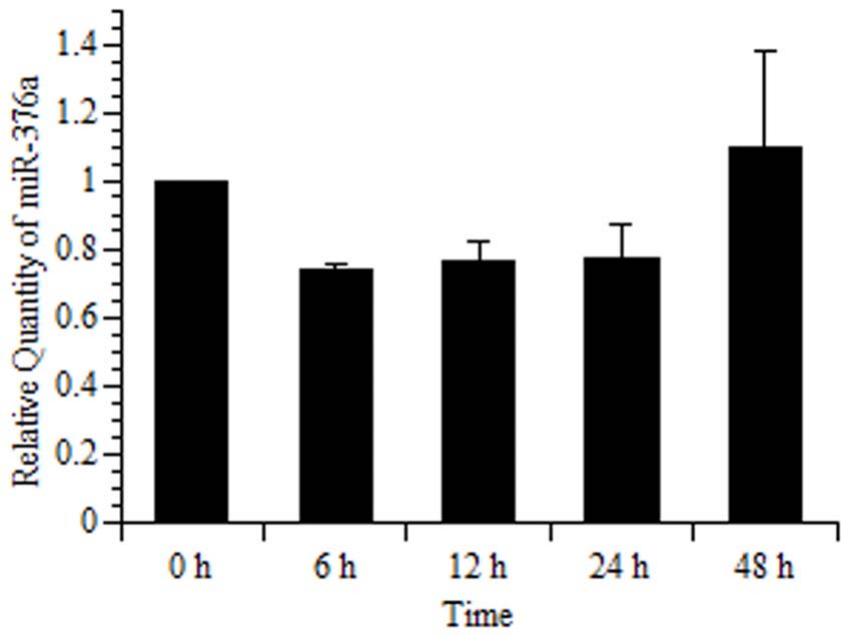
Rat GRP78 mRNA, rno-miR-376a expression in primary rat granulosa cells induced by FSH. Primary rat granulosa cells were prepared, and the indicated reagents were added to the medium after 24 h of culture. Cells were then incubated with FSH (30 ng/mL) and estradiol (10 nM) for 48 h in the same way as described in Fig. 2. The time after 48 h of incubation with FSH and estradiol was considered “0 h.” Total RNA was isolated, and rno-miR-376a expression levels was determined using real-time RT-PCR at the indicated time. The amounts of rno-miR-376a in the 0 h group were set at 1. Data were normalized for 4.5S RNA(H) levels in each sample and represent the mean ±SE of 3 independent experiments.

Next, we investigated the effects of rno-miR-144, rno-miR-376a, and rno-miR-451 on GRP78 mRNA expression in granulosa cells isolated from DES-treated immature rats (Fig. 2). GRP78 mRNA and rno-miR-376a expression increased significantly 12 h after the addition of hCG into the culture medium and subsequently decreased, which was consistent with *in vivo* study (Fig. 1). In contrast, rno-miR-144 and rno-miR-451 expression decreased significantly after the hCG treatment. Furthermore, miR-376a did not show a substantial change in the absence of hCG treatment (Fig. 3), confirming that hCG has an important role for the induction of miR-376a.

### miR-376a does not affect GRP78 mRNA expression

**Figure 4:**
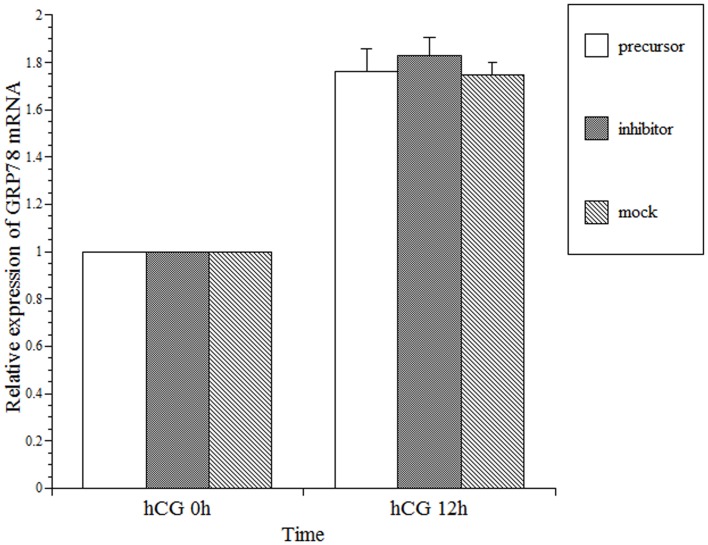
Effects of Pre-miR-376a (precursor) and Anti-miR-376a (inhibitor) transfection on rat GRP78 mRNA expression in primary rat granulosa cells. Primary rat granulosa cells were prepared, and the indicated reagents were added to the medium after 24 h of culture. The cells were incubated with FSH (30 ng/mL) and estradiol (10 nM) for 48 h. Pre-miR-376a (precursor) or Anti-miR-376a (inhibitor) was transfected into the cells, and 30 ng/mL hCG was added 12 h later. The effects of precursor and inhibitor on the expression of GRP78 mRNA were measured using real-time RT-PCR, as described in the Materials and Methods. The expression of GRP78 mRNA at hCG 0 h in the control was set at 1. Each value represents the mean ±SE of three independent experiments.

We then examined the effects of rno-miR-376a on GRP78 mRNA levels following the transfection of granulosa cells with either precursor or inhibitor (Fig. 4). In this experiment, the granulosa cells were cultured in a similar manner to the cells utilized in Fig. 2 except that the cells were transfected with precursor or inhibitor 12 h prior to hCG treatment. The results indicated that the transfection with precursor or inhibitor did not affect GRP78 mRNA expression.

### miR-376a represses GRP78 translation

**Figure 5:**
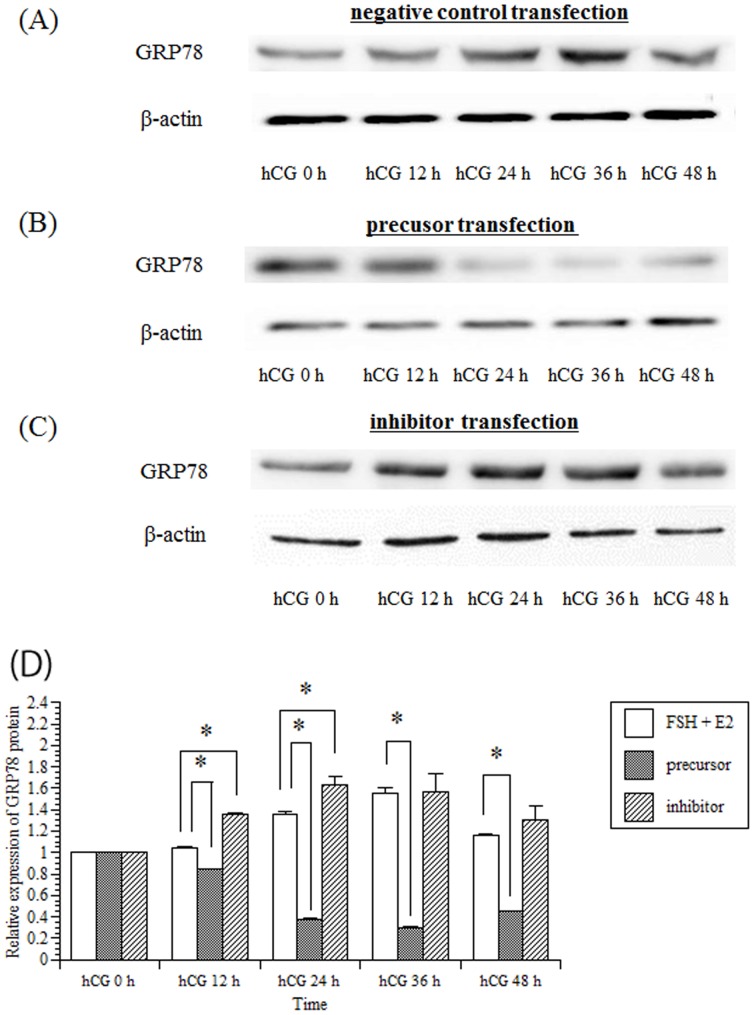
Effects of rno-miR-376a on GRP78 protein in granulosa cells. Primary rat granulosa cells were prepared, and the indicated reagents were added to the medium after 24 h of culture. The cells were incubated with FSH (30 ng/mL) and estradiol (10 nM) for 48 h. Negative control (A), precursor (B) or inhibitor (C) was transfected into the cells, and 30 ng/mL hCG was added 12 h later. Cells were harvested 24 h after the addition of hCG, and GRP78 protein levels were quantified using western blot analysis. (D) Levels of GRP78 protein were quantified by densitometric scanning. The expression of GRP78 protein in the control (hCG 0 h) was set at 1. Values represent the mean ±SE of three independent experiments. *, significantly different from the control value, *P*<0.05.

Furthermore, to ascertain the expression of GRP78 protein, western blot analysis (Fig. 5) was performed using the same animal model designed for the real-time RT-PCR analysis. The results indicated that the transfection of the granulosa cells with precursor significantly decreased GRP78 protein (Fig. 5B). However, the transfection of granulosa cells with inhibitor upregulated GRP78 protein expression 24 h after the hCG treatment (Fig. 5C).

### miR-376a binds to predicted site of GRP78 3′-UTR

**Figure 6:**
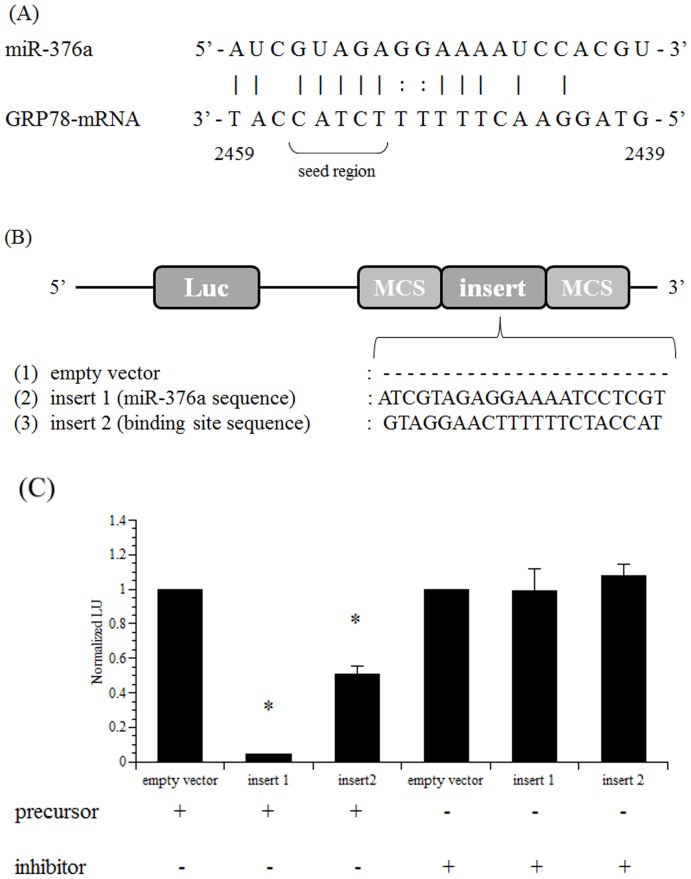
Luciferase assays for the identification of the rno-miR-376a-binding site in the 3′-UTR of GRP78 mRNA. (A) Arrangement of rno-miR-376a and GRP78 mRNA and a schematic drawing of the predicted rno-miR-376a-binding site in the 3′-UTR of GRP78 mRNA. (B) Schematic drawings of the pMIR-REPORT luciferase vectors used in our experiment. To identify the rno-miR-376a-binding site in the 3′-UTR of GRP78 mRNA, luciferase reporter vectors were generated as described in the Materials and Methods. (C) Luciferase activity was measured to identify the rno-miR-376a-binding site in the 3′-UTR of GRP78 mRNA. HEK293 cells were prepared, and the cells were transfected with 200 ng of each reporter vector with 50 nM Pre-miR-376a (precursor) or Anti-miR-376a (inhibitor) as described in the Materials and Methods. For transfection normalization, the cells were also transfected with the pMIR-REPORT βgal vector. Luciferase activity was measured 24 h after transfection. The activity of the control (empty vector) was set at 1. Each value represents the mean ±SE of three independent experiments. *, significantly different from the control value, *P*<0.05.

To confirm the presence of an rno-miR-376a binding site on GRP78 mRNA, we constructed a reporter vector that contained a putative rno-miR-376a binding site sequence (bp 2439–2459) in the 3′-UTR downstream of a *Renilla* luciferase coding region. To avoid the influence of endogenous rno-miR-376a, we used HEK293 cells rather than rat granulosa cells (Fig. 6). To verify the effect of the miRNA transfection, we generated a reporter vector that included the whole miR-376a sequence. The results indicated that precursor reduced the luciferase activity to approximately 0%, while inhibitor did not affect the luciferase activity. These data demonstrate that the translation of GRP78 was blocked by precursor via a binding site that was the complementary sequence to rno-miR-376a.

The luciferase activity in the cells transfected with the reporter vector containing the putative rno-miR-376a binding site was reduced by approximately 50% 24 h after the cells were transfected with precursor. In contrast, the transfection with inhibitor did not affect the luciferase activity. This result indicates that rno-miR-376a binds to the 3′-end of GRP78 mRNA from bp 2439–2459 in the 3′-UTR.

## Discussion

The transition from the ovulatory follicle to corpus luteum formation is a critical phase for maintaining female reproductive functions. Within a follicle after ovulation, many different cell types are subjected to dramatic changes, including proliferation, apoptosis, and differentiation. Thus, the ovulatory stimuli provided by the LH surge trigger the synthesis of numerous proteins inside cells, indicating that ER-associated chaperones facilitate proper maturation of newly synthesized proteins. In fact, our recent finding suggests that GRP78 is involved in restoring LHR expression following the down-regulation induced by ovulation [10]. Therefore, to better understand the regulation of GRP78 expression, we focused on miRNAs that are induced in the ovary after ovulation because increasing evidence suggests that miRNAs play pivotal roles in the regulation of a wide range of biological processes [18], [20], [21].

To identify miRNAs that potentially regulate GRP78 expression, microRNA microarrays were performed using ovaries from PMSG-primed rats injected with an ovulatory dose of hCG. The array data along with the bioinformatic analysis provided by MicroCosm Targets, which indicated that several miRNAs bind to the GRP78 mRNA 3′-UTR, led us to focus on rno-miR-144, rno-miR-376a, and rno-miR-451. From these, we narrowed the focus to rno-miR-376a based on the results of the *in vitro* experiments (Fig. 2) since rno-miR-144 and rno-miR-451 was not induced expression by hCG treatment. We believe that the discrepancy regarding in the expression of miR-144 and 451 between in the *in vivo* and *in vitro* experiments (Fig. 1 and 2) can be explained by the presence of blood cells in the *in vivo* study which express both rno-miR-144 and rno-miR-451 [22], [23].

Initially, miRNAs were thought to bind within the 3′-UTR of their target mRNAs, but recent evidence has demonstrated that the 5′-UTR [24] or coding region [25] can also be the target sites for miRNAs. As more than 60% of human protein coding genes are predicted to be under the control of miRNAs by binding within their 3′-UTR [26], [27], GRP78 mRNA in the rat ovary is regulated post-transcriptionally by miRNAs in a same manner as the majority of human mRNAs.

Predicting miRNA targets using computational analysis, which was employed for this study, is efficient but can result in tens or hundreds of targets with high false-positive rates [28]. To avoid this issue, we executed a luciferase assay to identify an rno-miR-376a-binding site in the 3′-end of GRP78 mRNA, which confirmed that the 3′-end of GRP78 mRNA from bp 2439 to 2459 is the binding site for rno-miR-376a. As mentioned above, miRNAs mainly control their target genes through base pairing to the 3′-UTRs of target mRNAs, resulting in the repression of translation of target proteins or the degradation of target mRNAs. In initial reports, the extent of complementarity between miRNA and its target mRNA is thought to govern either translational mRNA cleavage or repression [29]: in plants, nearly perfect complementarity results in the degradation of target miRNAs [30], whereas in animals, partial complementarity results in a translational block [31]. However, recent studies have reported that miRNAs can also induce mRNA degradation in animals and, conversely, translational repression in plants, although the topic remains controversial due to conflicting data [32], [33]. Moreover, the state of the complementarity between target mRNA and miRNA has been suggested to affect mRNA degradation [34], which is applicable to rno-miR-376a because the introduction of the complete identical sequence of rno-miR-376a into a reporter vector abolished the luciferase activity, whereas the luciferase activity was reduced to 50% using the reporter vector containing the 3′-UTR rno-miR-376a binding site of GRP78 mRNA (Fig. 6).

The results presented in this study (Fig. 4 and Fig. 5) demonstrate that rno-miR-376a decreases GRP78 protein production by translational repression without altering GRP78 mRNA levels and that the transfection of rno-miR-376a into granulosa cells repressed protein expression by approximately three-fold (Fig. 5). One can argue whether this reduction of GRP78 protein by rno-miR-376a has significant meaning for physiological functions in the ovary. In our previous report [10], three-fold reduction in the level of GRP78 protein significantly impaired restoration of LHR expression in the granulosa cells after the LHR down-regulation induced by ovulation. In addition, previous reports have concluded that the magnitude of repression by miRNA rarely exceeds two-fold [26], [35]. Thus, we believe that rno-miR-376a is involved in the regulation of GRP78 expression in the ovary.

It has been accepted that a single miRNA can downregulate hundreds of mRNAs and the production of hundreds of proteins [26], [36]. Combined with the MicroCosm analysis of miRNA targets, which revealed that 44 miRNAs can bind to the 3′-UTR of GRP78 mRNA, we speculate that multiple miRNAs may constitute a network that is involved in the regulation of GRP78 mRNA, whereas rno-miR-376a is solely identified to be evoked by the ovulatory signal. In the regulation of gene expression, many intermediate steps occur during the processing of RNA to protein. GRP78, which is regarded as a stress-inducible house-keeping gene, is transcribed constitutively. Therefore, the post-transcriptional control of GRP78 by rno-miR-376a has a significant impact on the regulation of GRP78 protein expression and not gene expression, which is evident by the lack of GRP78 transcriptional regulation.

In conclusion, the present results demonstrate that the induction of rno-miR-376a production by hCG leads to repression of GRP78 translation. Although the precise mechanism regulating GRP78 has not been elucidated, our finding may provide clues for those investigating miRNA-mediated regulation of ovarian functions.

## Supporting Information

Figure S1 **Hierarchical clustering analysis of miRNA expression in rat ovaries induced PMSG and hCG.** The clustering analysis was conducted by TaKaRa Bio Inc. and was carried out using the complete linkage method. Forty-four differentially expressed miRNAs chosen with an adjusted false discovery rate <0.05. Each row represents an individual miRNA and each column represents the time point after the injection of hCG. The miRNA clustering tree is shown on the left. The color scale illustrates the relative expression level of miRNAs.
(TIF)

## References

[1] Zeleznik AJ, Midgley AR Jr, Reichert LE Jr (1974) Granulosa cell maturation in the rat: increased binding of human chorionic gonadotropin following treatment with follicle-stimulating hormone in vivo. Endocrinology 95: 818–825. 4368756 10.1210/endo-95-3-818

[2] Zeleznik AJ, Schuler HM, Reichert LE Jr (1981) Gonadotropin-binding sites in the rhesus monkey ovary: role of the vasculature in the selective distribution of human chorionic gonadotropin to the preovulatory follicle. Endocrinology 109: 356–362. 6265188 10.1210/endo-109-2-356

[3] Menon KM, Clouser CL, Nair AK (2005) Gonadotropin receptors: role of post-translational modifications and post-transcriptional regulation. Endocrine 26: 249–257. 16034179 10.1385/ENDO:26:3:249

[4] Espey LL, Richards JS (2002) Temporal and spatial patterns of ovarian gene transcription following an ovulatory dose of gonadotropin in the rat. Biol Reprod 67: 1662–1670. 12444039 10.1095/biolreprod.102.005173

[5] Richards JS, Russell DL, Ochsner S, Espey LL (2002) Ovulation: new dimensions and new regulators of the inflammatory-like response. Annu Rev Physiol 64: 69–92. 11826264 10.1146/annurev.physiol.64.081501.131029

[6] Lee AS (2001) The glucose-regulated proteins: stress induction and clinical applications. Trends Biochem Sci 26: 504–510. 11504627 10.1016/s0968-0004(01)01908-9

[7] Klausner RD, Sitia R (1990) Protein degradation in the endoplasmic reticulum. Cell 62: 611–614. 2201450 10.1016/0092-8674(90)90104-m

[8] Bergeron JJ, Brenner MB, Thomas DY, Williams DB (1994) Calnexin: a membrane-bound chaperone of the endoplasmic reticulum. Trends Biochem Sci 19: 124–128. 8203019 10.1016/0968-0004(94)90205-4

[9] Ruddon RW, Bedows E (1997) Assisted protein folding. J Biol Chem 272: 3125–3128. 9081984 10.1074/jbc.272.6.3125

[10] Kogure K, Nakamura K, Ikeda S, Kitahara Y, Nishimura T, et al (2013) Glucose-regulated protein, 78-kilodalton is a modulator of luteinizing hormone receptor expression in luteinizing granulosa cells in rats. Biol Reprod 88: 8. 23175774 10.1095/biolreprod.112.101873

[11] Krol J, Loedige I, Filipowicz W (2010) The widespread regulation of microRNA biogenesis, function and decay. Nat Rev Genet 11: 597–610. 20661255 10.1038/nrg2843

[12] Wang Y, Lee CG (2009) MicroRNA and cancer–focus on apoptosis. J Cell Mol Med 13: 12–23. 19175697 10.1111/j.1582-4934.2008.00510.x PMC3823033

[13] Lynam-Lennon N, Maher SG, Reynolds JV (2009) The roles of microRNA in cancer and apoptosis. Biol Rev Camb Philos Soc 84: 55–71. 19046400 10.1111/j.1469-185X.2008.00061.x

[14] Bueno MJ, Perez de Castro I, Malumbres M (2008) Control of cell proliferation pathways by microRNAs. Cell Cycle 7: 3143–3148. 18843198 10.4161/cc.7.20.6833

[15] Carletti MZ, Fiedler SD, Christenson LK (2010) MicroRNA 21 blocks apoptosis in mouse periovulatory granulosa cells. Biol Reprod 83: 286–295. 20357270 10.1095/biolreprod.109.081448 PMC2907287

[16] Yao G, Yin M, Lian J, Tian H, Liu L, et al (2010) MicroRNA-224 is involved in transforming growth factor-beta-mediated mouse granulosa cell proliferation and granulosa cell function by targeting Smad4. Mol Endocrinol 24: 540–551. 20118412 10.1210/me.2009-0432 PMC5419098

[17] Xu S, Linher-Melville K, Yang BB, Wu D, Li J (2011) Micro-RNA378 (miR-378) regulates ovarian estradiol production by targeting aromatase. Endocrinology 152: 3941–3951. 21846797 10.1210/en.2011-1147 PMC3176644

[18] Otsuka M, Zheng M, Hayashi M, Lee JD, Yoshino O, et al (2008) Impaired microRNA processing causes corpus luteum insufficiency and infertility in mice. J Clin Invest 118: 1944–1954. 18398510 10.1172/JCI33680 PMC2289794

[19] Kitahara Y, Nakamura K, Kogure K, Minegishi T (2013) Role of microRNA-136-3p on the Expression of Luteinizing Hormone-Human Chorionic Gonadotropin Receptor mRNA in Rat Ovaries. Biol Reprod 89: 114. 24025743 10.1095/biolreprod.113.109207

[20] Lin F, Li R, Pan ZX, Zhou B, Yu de B, et al (2012) miR-26b promotes granulosa cell apoptosis by targeting ATM during follicular atresia in porcine ovary. PLoS One 7: e38640. 22737216 10.1371/journal.pone.0038640 PMC3380909

[21] McBride D, Carre W, Sontakke SD, Hogg CO, Law A, et al (2012) Identification of miRNAs associated with the follicular-luteal transition in the ruminant ovary. Reproduction 144: 221–233. 22653318 10.1530/REP-12-0025

[22] Sangokoya C, Telen MJ, Chi JT (2010) microRNA miR-144 modulates oxidative stress tolerance and associates with anemia severity in sickle cell disease. Blood 116: 4338–4348. 20709907 10.1182/blood-2009-04-214817 PMC2993631

[23] Pritchard CC, Kroh E, Wood B, Arroyo JD, Dougherty KJ, et al (2012) Blood cell origin of circulating microRNAs: a cautionary note for cancer biomarker studies. Cancer Prev Res (Phila) 5: 492–497. 22158052 10.1158/1940-6207.CAPR-11-0370 PMC4186243

[24] Lytle JR, Yario TA, Steitz JA (2007) Target mRNAs are repressed as efficiently by microRNA-binding sites in the 5′ UTR as in the 3′ UTR. Proc Natl Acad Sci U S A 104: 9667–9672. 17535905 10.1073/pnas.0703820104 PMC1887587

[25] Forman JJ, Legesse-Miller A, Coller HA (2008) A search for conserved sequences in coding regions reveals that the let-7 microRNA targets Dicer within its coding sequence. Proc Natl Acad Sci U S A 105: 14879–14884. 18812516 10.1073/pnas.0803230105 PMC2567461

[26] Selbach M, Schwanhausser B, Thierfelder N, Fang Z, Khanin R, et al (2008) Widespread changes in protein synthesis induced by microRNAs. Nature 455: 58–63. 18668040 10.1038/nature07228

[27] Friedman RC, Farh KK, Burge CB, Bartel DP (2009) Most mammalian mRNAs are conserved targets of microRNAs. Genome Res 19: 92–105. 18955434 10.1101/gr.082701.108 PMC2612969

[28] Seitz H (2009) Redefining microRNA targets. Curr Biol 19: 870–873. 19375315 10.1016/j.cub.2009.03.059

[29] Bartel DP (2004) MicroRNAs: genomics, biogenesis, mechanism, and function. Cell 116: 281–297. 14744438 10.1016/s0092-8674(04)00045-5

[30] Palatnik JF, Allen E, Wu X, Schommer C, Schwab R, et al (2003) Control of leaf morphogenesis by microRNAs. Nature 425: 257–263. 12931144 10.1038/nature01958

[31] Chen X (2004) A microRNA as a translational repressor of APETALA2 in Arabidopsis flower development. Science 303: 2022–2025. 12893888 10.1126/science.1088060 PMC5127708

[32] Wu L, Belasco JG (2008) Let me count the ways: mechanisms of gene regulation by miRNAs and siRNAs. Mol Cell 29: 1–7. 18206964 10.1016/j.molcel.2007.12.010

[33] Carthew RW, Sontheimer EJ (2009) Origins and Mechanisms of miRNAs and siRNAs. Cell 136: 642–655. 19239886 10.1016/j.cell.2009.01.035 PMC2675692

[34] Ameres SL, Horwich MD, Hung JH, Xu J, Ghildiyal M, et al (2010) Target RNA-directed trimming and tailing of small silencing RNAs. Science 328: 1534–1539. 20558712 10.1126/science.1187058 PMC2902985

[35] Baek D, Villen J, Shin C, Camargo FD, Gygi SP, et al (2008) The impact of microRNAs on protein output. Nature 455: 64–71. 18668037 10.1038/nature07242 PMC2745094

[36] Lim LP, Lau NC, Garrett-Engele P, Grimson A, Schelter JM, et al (2005) Microarray analysis shows that some microRNAs downregulate large numbers of target mRNAs. Nature 433: 769–773. 15685193 10.1038/nature03315

