# Supplementary figures and images for: MicroRNA-376a Regulates 78-Kilodalton Glucose-Regulated Protein Expression in Rat Granulosa Cells
Source: PLoS One. 2014 Oct 3;9(10):e108997. doi: 10.1371/journal.pone.0108997 (PMC4184830; doi:10.1371/journal.pone.0108997)

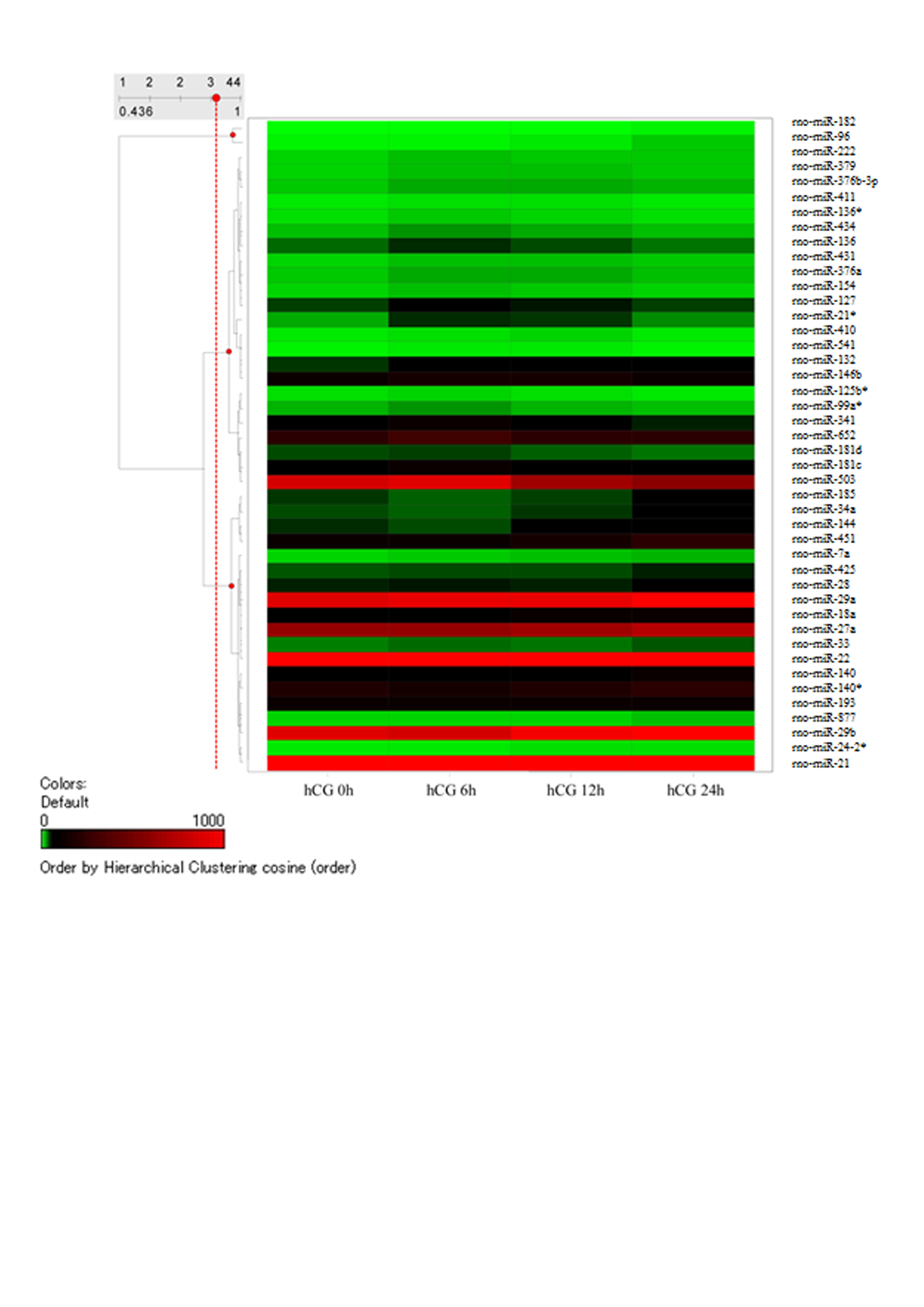

Supplement: Figure S1 — Hierarchical clustering analysis of miRNA expression in rat ovaries induced PMSG and hCG. The clustering analysis was conducted by TaKaRa Bio Inc. and was carried out using the complete linkage method. Forty-four differentially expressed miRNAs chosen with an adjusted false discovery rate <0.05. Each row represents an individual miRNA and each column represents the time point after the injection of hCG. The miRNA clustering tree is shown on the left. The color scale illustrates the relative expression level of miRNAs. (TIF) [file pone.0108997.s001.tif]
